# Isolated Nocardiosis, an Unrecognized Primary Immunodeficiency?

**DOI:** 10.3389/fimmu.2020.590239

**Published:** 2020-10-20

**Authors:** Rubén Martínez-Barricarte

**Affiliations:** ^1^Division of Genetic Medicine, Department of Medicine, Vanderbilt Genetics Institute, Vanderbilt University Medical Center, Nashville, TN, United States; ^2^Department of Pathology, Microbiology, and Immunology, Vanderbilt Center for Immunobiology, Vanderbilt Institute for Infection, Immunology, and Inflammation, Vanderbilt University Medical Center, Nashville, TN, United States

**Keywords:** *Nocardia*, nocardiosis, infection, primary immunodeficiencies, immune response, isolated nocardiosis, PID

## Abstract

Nocardiosis is an infectious disease caused by the gram-positive bacterium *Nocardia* spp. Although it is commonly accepted that exposure to *Nocardia* is almost universal, only a small fraction of exposed individuals develop the disease, while the vast majority remain healthy. Nocardiosis has been described as an “opportunistic” disease of immunocompromised patients, suggesting that exposure to the pathogen is necessary, but a host predisposition is also required. Interestingly, increasing numbers of nocardiosis cases in individuals without any detected risk factors, i.e., without overt immunodeficiency, are being reported. Furthermore, a growing body of evidence have shown that selective susceptibility to a specific pathogen can be caused by a primary immunodeficiency (PID). This raises the question of whether an undiagnosed PID may cause nocardiosis affecting otherwise healthy individuals. This review summarizes the specific clinical and microbiological characteristics of patients with isolated nocardiosis published during the past 30 years. Furthermore, it gives an overview of the known human immune mechanisms to fend off *Nocardia* spp. obtained from the study of PIDs and patients under immunomodulatory therapies.

## Introduction

In 1888, while investigating a disease in cattle called “*francine du boeuf*,” the veterinarian Edmond Nocard identified a gram-positive, acid-fast agent as causative of this disease (1). A year after, Trevisan named this genus *Nocardia* in honor of Nocard (2). Shortly thereafter, Eppinger isolated from the brain abscess of a 52-year-old patient, an organism that produced star-shaped colonies when grown in agar. Although he called it *Cladothrix asteroides*, it was later found to be *Nocardia asteroides*. This finding made *Nocardia* be the first human pathogenic aerobic actinomycete described in the literature (3, 4). Since then and until the advent of antibiotics, over 30 cases of nocardiosis were described published, all but three with fatal outcome or very severe sequelae (5). After the generalization of antibiotic therapy, recovery rates improved to 54%, and an increased number of patients were every year (6). At this point, reports started to associate nocardiosis with underlying conditions. The first of such reports was published in 1954 in a patient with nocardiosis during Hodgkin's lymphoma (7). Many reports followed showing cases of nocardiosis in patients with alveolar proteinosis (8), prolonged steroid usage (9, 10), Cushing's syndrome (11), patients under immunosuppressive drugs after transplantation (12) or neoplastic disorders (6) amongst other pathologies (Table 1). Besides, reports started to be published describing severe nocardiosis in patients with primary immunodeficiencies (PID) such as hypogammaglobulinemia (13, 14), or chronic granulomatous disease (CGD) (15–17) (Table 2). At this point, nocardiosis was proposed to be an opportunistic infection (19–21). That notion was accepted in the mid-80s after a growing number of nocardiosis cases were described in patients with AIDS (22–24). Interestingly, epidemiological studies in the United States of America indicated that not all patients with nocardiosis suffer from underlying disease (25, 26). As a matter of fact, according to the Center for Disease Control (CDC), one-third of nocardiosis patients have no known underlying condition. Furthermore, a growing body of evidence shows that severe infectious diseases in otherwise healthy individuals can arise from single-gene inborn errors of immunity (27, 28). Some examples of this are mendelian susceptibility to mycobacterial disease (MSMD) (28), human papillomavirus-associated epidermodysplasia verruciformis (29), invasive pneumococcal disease (30), invasive dermatophytic disease (31), or chronic mucocutaneous candidiasis (32). All these genetic diseases confer a selective susceptibility to a specific pathogen of weak virulence and ubiquitous exposure, much-resembling nocardiosis in patients without identified underlying conditions. In this review, we describe the epidemiological, clinical, and microbiological characteristics of 400 patients with nocardiosis without identified risk factors published during the past 30 years. We have also reviewed what we have learnt from patients with PIDs and patients with different comorbidities about the critical players of human immunity to *Nocardia* spp.

**Table 1:** Some risk factors for nocardiosis.

**Diseases**
AIDS
Solid-organ transplant
Chronic obstructive pulmonary disease
Chronic kidney disease
Cushing's syndrome
Pulmonary fibrosis
Diabetes mellitus
Systemic Lupus erythematosus
Hematopoietic stem cell transplantation
Drug abuse
Malignancies
End-stage renal disease
Membranoproliferative glomerulonephritis
Lung sarcoidosis
Pulmonary proteinosis
Alcoholism long history of smoking

**Table 2:** PIDs that can cause nocardiosis.

**Disease**	**Immunological consequences**
CGD	Defects in the NADPH oxidase complex that impairs the capacity of phagocytes to produce reactive oxygen species.
Hypogammaglobulinemia	Reduction in the titers of circulating antibodies.
CVID	Deficient levels of IgG, IgA, and IgM^*^.
Hyper IgE syndrome	Elevated serum IgE level, chronic dermatitis, intense pruritus, and severe recurrent infection.
Idiopathic CD4^+^ T-lymphocytopenia	Low levels of CD4^+^ T cells.
SCID	Lack of B and T cells.
MSMD	IL-12 and IL-23 abolishment^**^.
Anti-GM-CSF autoantibodies	Blockade of GM-CSF.

* *CVID is a heterogeneous group of diseases that can present with multiple different immunological abnormalities. The immunological consequences shown here are the ones observed in the patient reported in Singh et al. (18)*.

** *The genetic causes of MSMD can impair multiple branches of IFN-γ-mediated immunity. The genetic etiologies of MSMD that sometimes curse with nocardiosis impair IL-12 and IL-23 signaling*.

## Epidemiology

**Figure 1:**
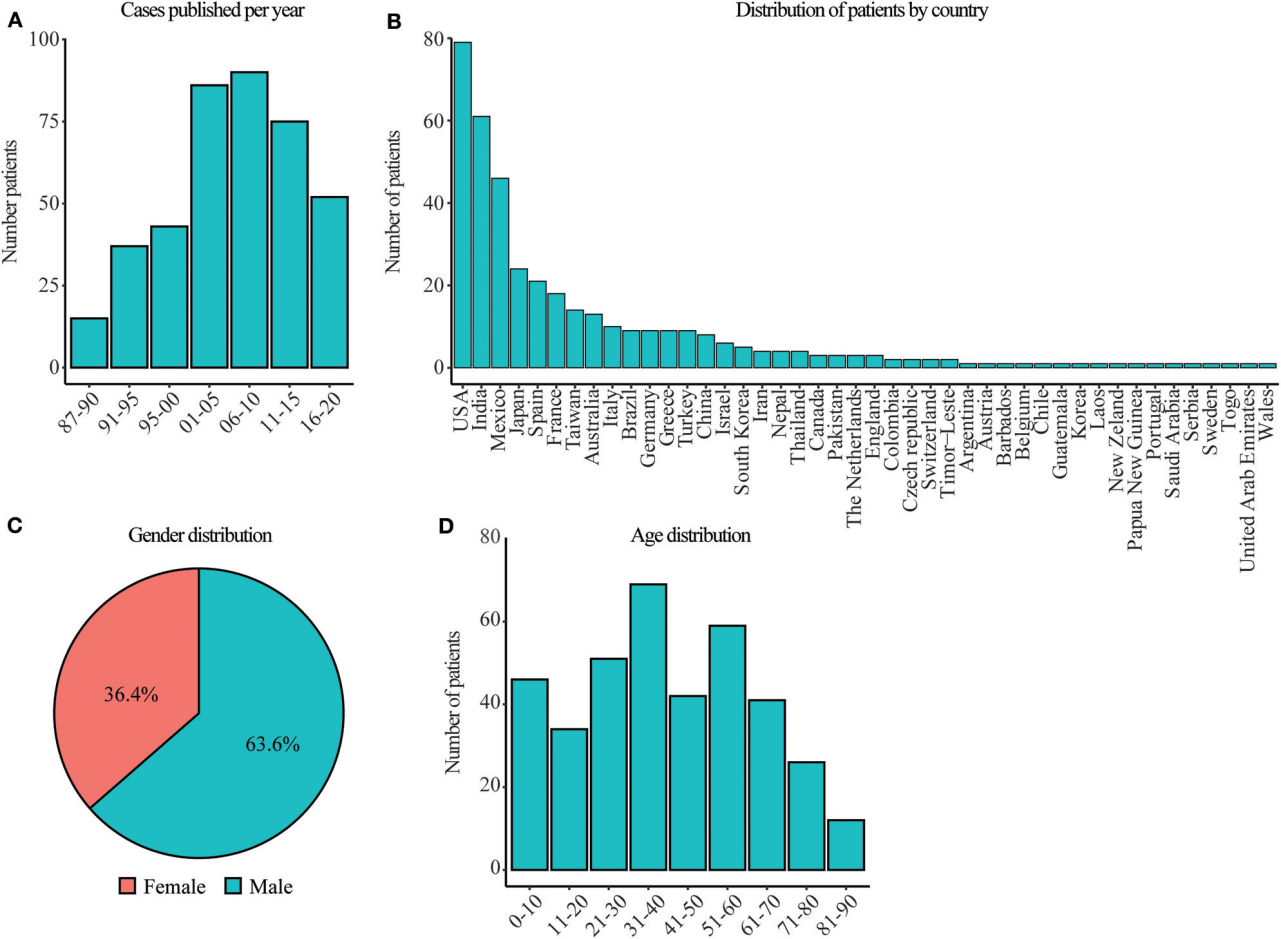
Date, gender, age, and geographical distribution of patients with isolated nocardiosis. **(A)** Year of publication of patients with isolated nocardiosis since 1987. Around 2010 the number of case series published increased in detriment of individual case reports. In most of those case series, we could not extract the data from individual patients so we excluded them from our analysis. Therefore, the number of cases continuously increases with time despite the graph shows a decrease after 2010. **(B)** Countries where patients from A were reported. **(C)** Gender distribution of patients with isolated nocardiosis. **(D)** Age distribution of patients with isolated nocardiosis published since 1987. The information for these figures was obtained from the references (33–297).

The description of patients with isolated nocardiosis after the HIV outbreak has been increasing steadily since the mid-80s, as evidenced by the number of case reports published in the literature over the last 30 years (Figure 1A). All the reported cases were sporadic, and no underlying condition was identified in any of them. Moreover, they did not come from consanguineous marriages, or the consanguinity was not indicated except in one patient (298). None of these patients were immunologically or genetically studied in search of a PID or other genetic defects. Patients with isolated nocardiosis have been found in 44 different countries spreading through 6 continents (all except Antarctica) with the USA, India, and Mexico having the most cases reported (Figure 1B). Contrary to other infectious diseases, nocardiosis is not a geographically confined disease, consistent with the fact that *Nocardia* spp. can be found throughout the world. We have observed that the number of males with isolated nocardiosis almost doubles that of female cases, similar to observations in immunocompromised individuals (25) (Figure 1C). Given that *Nocardia* spp. grow in soil; these gender biases may be due to a difference in exposure caused by the distinct lifestyle and profession of man vs. women. Besides, the female hormone estradiol has been shown to inhibit the growth of *Nocardia brasiliensis in vitro*, suggesting that the presence of this hormone might also contribute to the gender difference observed (299). Furthermore, this may be an indication of an undiagnosed X-linked immunodeficiency. We have found a homogeneous distribution among age groups in patients with isolated nocardiosis with a slight increase in the numbers of patients in the decades of 31–40 and 51–60 years of age (Figure 1D). This age distribution suggests that disease occurs when exposure to the pathogen happens thought life without any specific age group been overrepresented or more susceptible. Transmission of *Nocardia* spp. do not occur from person to person, and outbreaks are rare. Since the 80s, a handful of outbreaks have been reported in the UK, France, Japan, Germany, and the US. All in groups of patients interned in hospitals and with some level of immunosuppression (300–306). Therefore, this observation indicates that the leading risk factor in developing nocardiosis is the immune status of the host rather than the virulence of the different *Nocardia* strains.

## Clinical Presentation

**Figure 2:**
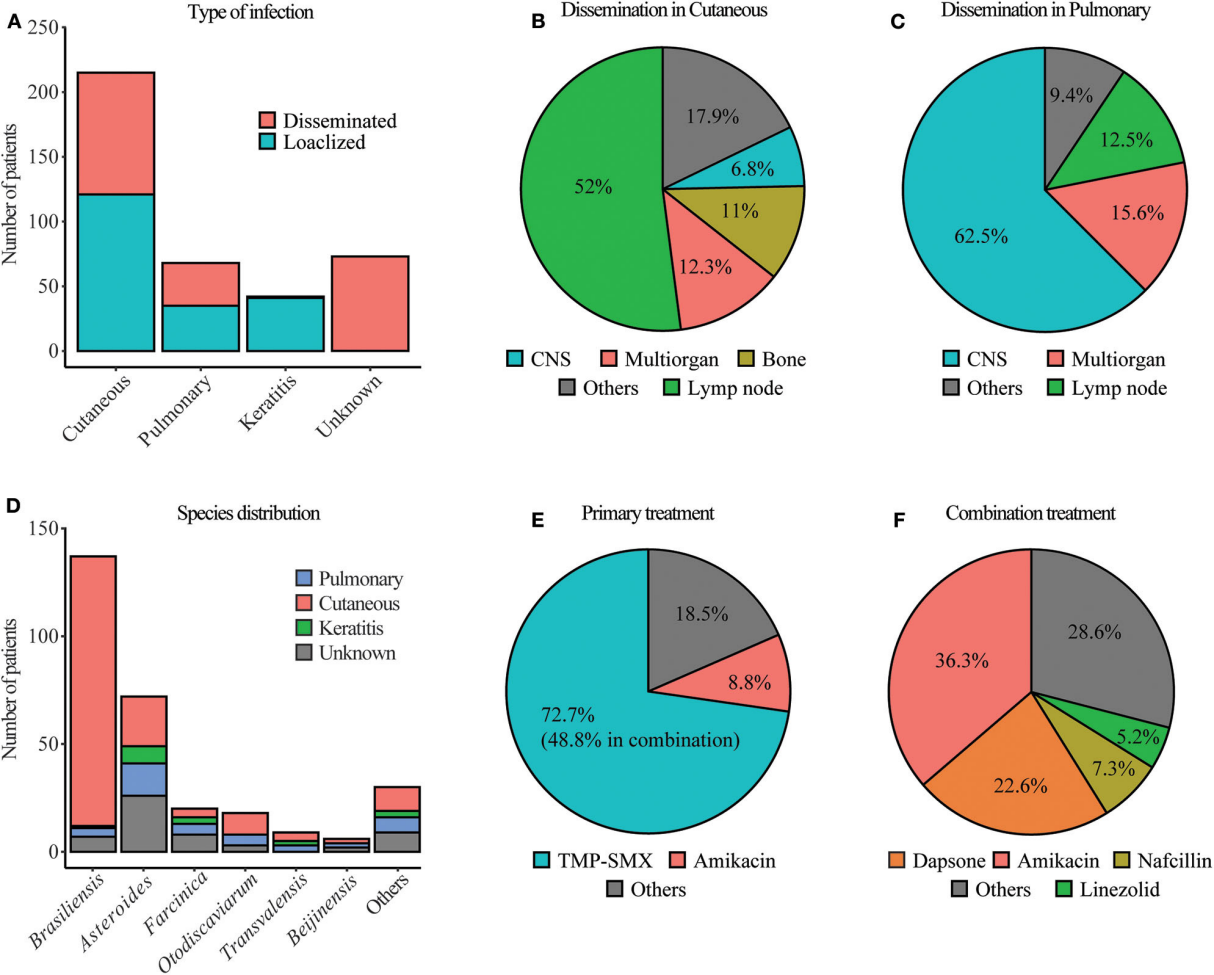
Microbiology, treatment, and kind of infection in patients with isolated nocardiosis. **(A)** Type of infection of patients with isolated nocardiosis published since 1987. Unknown represent patients with disseminated nocardiosis in which the primary site of infection was not identified. **(B,C)** Organs, where the disease disseminated in patients with cutaneous nocardiosis in **(B)** and pulmonary nocardiosis in **(C)**. Others, represent organs with a frequency of <5% in this group. **(D)** Distribution of *Nocardia* species in the different types of nocardiosis at a frequency of <5%. Others represent the species present. **(E)** Primary treatment of patients with isolated nocardiosis. **(F)** Therapy that was used in combination with TMP-SMX. Others represent the use of drugs at a frequency of <5%. The information for these figures was obtained from the references (33–297).

Nocardiosis can present as a cutaneous disease when the bacterium is inoculated in the skin, usually through a puncture or wound, pulmonary when the bacterium is inhaled and gets to the lungs or disseminated when from these initial foci of infection, it spreads to other organs. In most cases, upon infection, the immune system neutralizes this bacterium, and individuals remain asymptomatic or suffer from a mild self-resolving disease that frequently goes undiagnosed. The classical presentation of pulmonary nocardiosis includes cough, fever, dyspnea, fatigue, and chest pain (307). At the radiological level, it can present as a focal or multifocal disease with infiltrates (nodular or consolidated) and pleural effusion (308). In the case of cutaneous or subcutaneous nocardiosis, the lesions usually show expanding nodules, cellulitis, and ulcerative draining lesions. A fraction of cutaneous nocardiosis develops as mycetoma. These lesions are chronic, slow-progressing, and painless, usually in the lower limbs that present with tumefaction, destructive granuloma, deformity, subcutaneous nodules, and discharging sinuses that exude pus (309). In *a priori*, immunocompetent individuals, a sizable number of patients have keratitis caused by *Nocardia* spp. Keratitis usually happens after cornea injury in contact lenses wearers, causing the inoculation of the pathogen. It presents as an inflammatory eye condition, pain, photophobia, and visual impairment. If untreated or misdiagnosed, it might lead to endophthalmitis (309). From the primary foci of infection, the disease can disseminate hematogenously potentially to any organ in the body, and after dissemination occurs, mortality and morbidity increase dramatically. *Nocardia* spp. have a particular tropism for the CNS. When dissemination happens there, the disease tends to present as headache, seizures, mental status change, confusion, ataxia, or focal neurological deficit may appear. A brain abscess is usually visible by computed tomography (CT) or magnetic resonance imaging (MRI) scans (310). It has been proposed that pulmonary nocardiosis is a disease of immunocompromised individuals, while the cutaneous disease is observed in immunocompetent individuals (308). However, in-depth immunological and genetic studies of patients with a priori, no additional risk factors have not been performed, preventing us from concluding if these patients referred to in the literature as immunocompetent have an undiagnosed immunodeficiency. We observed that the majority of cases of isolated nocardiosis showed cutaneous disease (Figure 2A). However, we also found a sizeable number of patients with pulmonary nocardiosis without underlying conditions. In both cases of isolated nocardiosis, cutaneous, and pulmonary, dissemination from the primary foci of infection is about 45% (Figure 2A). Patients with keratitis caused by *Nocardia* spp., except in one case, do not present with disseminated disease. Besides, 15% of patients reported presented with a disseminated disease without identification of the primary location of the infection (Figure 2A). When dissemination happened, the organs to which the disease disseminated was dependent on the original site of infection. In more than half of the patients with dissemination from cutaneous infection, the organs affected were the lymph nodes closest to the location of infections. In 12% of the cases, the dissemination occurred to multiple organs, and in 11%, cutaneous nocardiosis gave rise to bone involvement, often osteomyelitis (Figure 2B). Other organs such as CNS, muscle, lung, salivary gland, or ovaries were involved in some patients. When dissemination occurred from primary pulmonary infection, 62% of the patients developed CNS nocardiosis, 15% suffered from multiorgan dissemination, and in 12%, the disease disseminated to the closest lymph nodes (Figure 2C). Other organs were also described to be affected in some patients, such as bone or mediastine.

## Microbiology

*Nocardia* is a genus of aerobic gram-positive bacteria of the order *Actinomycetales*. It is characterized by weak acid-fast stain, catalase-positive, presence of mycolic acid in its cell wall, and rod-shaped that grows in branches, sometimes confusing itself with fungi. It can be found in soil and areas rich in organic material. *Nocardia* has also been reported to be a commensal after it was identified in the skin, lung aspirates, and gingiva from healthy individuals (311–313), although this has recently been refuted (314). At the time of writing this report, there were 109 different *Nocardia* species with confirmed names according to the List of Prokaryotic names with Standing Nomenclature (LPSN) (https://www.bacterio.net). Among them, 54 had been reported to cause disease in humans (315). We have seen that 23 different species cause disease in otherwise healthy individuals (Table 3). Nowadays, the guidelines for identifying *Nocardia* up to the species without ambiguity suggest a combination of MALDI-TOF mass spectrometry, gene sequencing, and, ultimately, genome sequencing (315). Using these methods, during the last decades, what was thought to be an individual species, like *Nocardia asteroides*, has been found to be composed of different species to the point that *N. asteroides* is no longer a valid nomenclature (315, 316). Unfortunately, most diagnostic labs do not have these capabilities, so *N. asteroides* is still considered in the clinical setting.

**Table 3:** Count of the different *Nocardia* species identified in patients with isolated nocardiosis.

**Pathogen**	***n***
*N. brasiliensis*	137
*N. asteroides*	68
*N. farcinica*	20
*N. otitidiscaviarum*	18
*N. transvalensis*	9
*N. beijingensis*	6
*N. nova*	5
*N. abscessus*	5
*N. wallacei*	3
*N. paucivorans*	3
*N. takedensis*	2
*N. elegans*	2
*N. cyriacigeorgica*	2
*N. asiatica*	2
*N. yamanashiensis*	1
*N. vinacea*	1
*N. puris*	1
*N. pseudobrasiliensis*	1
*N. harenae*	1
*N. crassostrea*	1
*N. caviae*	1
*N. arthritidis*	1
*N. aobensis*	1

## Diagnosis

Multiple proteins from *N. asteroides* and *brasiliensis* have been described as being specifically recognized by patients who have suffered nocardiosis but not by healthy controls (317–321). These serologic tests are not commercially available, and the ones described are unreliable, making clinical diagnosis difficult (322). The observation of the symptoms and other physical tests like radiologic images may be suggestive of the disease (322). However, observation of *Nocardia* in cultures is necessary to confirm the diagnosis (309). Identification of the pathogen occurs by growing the clinical specimen (sputum, bronchoalveolar lavage, pleural effusion, pus) on plates like tryptic soy blood agar at 35°C. The identification of *Nocardia* is usually based on partial acid-fastness, resistance to lysozyme, and presence of chalky white colonies, dichotomous branches, and aerial hyphae (323). Colonies may appear after 2 days, but it is recommended to keep the plates in culture for up to 3 weeks since many species take longer to grow. Therefore, the prolonged incubation required to identify the bacteria that, unfortunately, many laboratories fail to follow, makes nocardiosis and underrepresented and underdiagnosed disease, especially in patients without risk factors. Despite the methodologies described above to identify *Nocardia* up to the specie, most diagnostic laboratories only have access to conventional biochemical tests such as tyrosine, xanthine, and hypoxanthine decomposition, growth at 45°C, and gelatin hydrolysis (323). The most used molecular biology methods that are found in a diagnostic lab to identify different *Nocardia* spp. are gene sequencing of 16S rRNA or hsp65. With these limitations in mind and considering that some patients described in this review were diagnosed up to 35 years ago, the most frequently identified species in patients with isolated nocardiosis are; *N. brasiliensis, N. asteroids, N. farcinica, N. otodiscaviarum, N. transvalensis* and *N. Beijinensis* (Figure 2D) (Table 3). Around 90% of cases of *N. brasiliensis* are cutaneous, consistent with what was observed in immunocompromised patients (324). However, the other five species cause cutaneous, pulmonary, or keratitis, with frequencies proportional to their identification in clinical specimens (Figure 2D). This contradicts the notion observed in immunocompromised individuals suggesting that *N. brasiliensis* causes cutaneous disease while *N. asteroides* causes pulmonary disease (324). The lack of clinical, epidemiological, and laboratory data undermines the significance of *Nocardia* spp. as a potential pathogen and causes a delay in the start of appropriate treatment (325). Sometimes the diagnosis only happens post mortem, highlighting the importance and necessity of improving the diagnosis methodologies and better understanding the causes of nocardiosis in these patients without risk factors (326).

## Treatment and Outcome

Treatment for nocardiosis has been evolving since the early 20th century. The first successful treatment of a patient with nocardiosis was described in 1907 by Musgrave and Clegg in a 30-year-old woman with mycetoma. Treatment consisted of amputating the affected extremity (327). Since then, only two more patients were successfully treated until the 40s (328, 329). After 1943, an increasing number of survivors were reported due to the use of the first antibiotics (sulfanilamide, penicillin, streptomycin, aureomycin), sulfonamides, and surgical incision and drainage (5). Until sulfonamide therapy became available, the mortality observed in patients with nocardiosis was 76%. After its implementation, mortality dropped to 46% (6). Nowadays, sulfonamides are the drug of choice, despite the first attempt at using them to treat nocardiosis in 1937 was unsuccessful (330). The first successful treatment of nocardiosis with sulfonamides happened a few years after in 1943 (331). In the mid-70s, patients started to be successfully treated with a 1:5 proportion of the dihydrofolate reductase inhibitor trimethoprim and the sulfonamide antibiotic sulfamethoxazole. This combination of drugs is known as TMP-SMX or co-trimoxazole. TMP-SMX has the advantage that it easily reaches antibacterial concentrations in the lung, blood, and central nervous system (332). Despite the question of whether TMP-SMX treatment was more effective than sulfonamides was controversial (333), it became the treatment of choice. It was thought that all *Nocardia* strains are susceptible to these drugs (334); however, this is not the case, and susceptibility testing should be performed before starting the treatment (335), given that resistance is specie specific (336, 337). Besides, some people are allergic to TMP-SMX, which is sometimes observed after treatment starts with the additional risk that this brings for the patient (338). The standards dictate that for cutaneous disease, TMP-SMX should be administered for 1–3 months, for pulmonary disease during 6–12 months, and for disseminated, especially CNS infection, it should be of 12 months sometimes, even more, depending on the progress of the disease (339). The long treatment necessary to cure nocardiosis with TMP-SMX highlights the commonly induced side effects such as nausea, vomiting, diarrhea, gingival hyperplasia (340), acute pancreatitis (341), myelosuppression, hepatoxicity, and renal insufficiency (308, 338) among others. Seventy-two percent of patients with isolated nocardiosis were treated with TMP-SMX, 8% with the aminoglycoside antibiotic, Amikacin, and the last 18% with other drugs such as the beta-lactam antibiotic imipenem or the penicillin antibiotic nafcillin among others (Figure 2E). In some cases, the treatment occurred only with one drug; however, about half of the patients treated with TMP-SMX had this treatment combined with other medications. This combination therapy involved Amikacin in 36% of the cases, dapsone in 22%, the beta-lactam antibiotic Nafcillin in 7% of the cases, and the oxazolidinone Linezolid in 5% of cases (Figure 2F). The way of administration of the drug varied depending on the kind of disease. In cutaneous or keratitis patients, the treatment was administered mostly topically, while in pulmonary or disseminated, the antibiotics were ingested. In the most severe cases, the medication was injected intravascularly. Antibiotic treatment was used in combination with surgical intervention in 18.3% of cases. Most surgeries consisted of debridement or drainage of affected areas in the lymph nodes, CNS, lungs, or extremities, although other interventions such as limb amputation were observed. Despite treatment, in 23.1% of patients, the disease could not be cured, becoming chronic, the disease recurred after the first episode was thought to be resolved, or the patients had sequelae such as impaired vision, neurological impairment, limb amputation, or severe scarring. Besides, an overall 5.7% of the patient died due to complications caused by nocardiosis. The outcome of these patients gives an idea of the severity of this disease even with appropriate treatment in patients that do not have any additional comorbidity. It also showcases the importance of early diagnosis and the need for better diagnostic and treatment options of isolated nocardiosis.

## Human Immunity to *Nocardia* spp.

Reports investigating how the human immune system fights *Nocardia* spp. are scarce. However, the study of patients treated with immunosuppressive drugs, anti-cytokine antibody, and information obtained from patients with PIDs has given us a clue of how such immunity is orchestrated (Tables 2, 4). PIDs are a group of diseases caused by single-gene inborn errors of immunity that impair specific cell-types or pathways of the immune system and are characterized by severe infection. The study of these diseases carried out during the last few decades has allowed us to delineate what are the critical and non-redundant cell-types and pathways in immunity against given pathogens (342). By analyzing what PIDs curse with nocardiosis, we can infer some of these anti-*Nocardia* immune mechanisms. The first PID in which nocardiosis was observed was chronic granulomatous disease (CGD). CGD is characterized by the impaired capacity of phagocytes to produce reactive oxygen species and, therefore, kill certain bacteria, especially catalase-positive ones (343). Early after the description of CGD, pulmonary nocardiosis started to be identified as one of the common infections suffered by these groups of patients (15–17). Since then, numerous reviews and cohort studies have shown the presence of nocardiosis among patients with CGD (343–347). From these groups of PID patients, we have been able to infer that phagocytes play a critical role in human immunity to *Nocardia* spp. Furthermore, it has been shown that human-derived alpha-defensins from neutrophils can kill certain strains of *Nocardia*, including *farcinica, nova*, and *asteroides* strengthening the evidence of the role of neutrophils in anti-*Nocardia* immunity (348).

**Table 4:** Treatments that affect the immune system and predispose to nocardiosis.

**Treatment**	**Immunological consequence**	**Examples of diseases treated**
Anti-BTK	Reduces B cell activation	Hematologic malignancies such aschronic lymphocytic leukemia (CLL).
Anti-CD3	Reduces T cell activation	Solid organ transplant rejection.
Anti-CTLA-4	Enhances T cell immunity	Melanoma, carcinoma.
Anti-CD52	Depletes peripheral blood lymphocytes	Multiple sclerosis, CLL.
Steroid	Reduces cellular immunity	Organ transplant rejection, asthma, allergies, dermatitis, Crohn's disease,rheumatoid arthritis.
Anti-TNF	Blocks of TNF signaling	Inflammatory bowel disease, Crohn's disease, rheumatoid arthritis.
Anty-IL12p40	Blocks IL-12 and IL-23 signaling	Inflammatory bowel disease, Crohn's disease, psoriasis.
Chemotherapy	Affects the bone marrow reducing cellular immunity	Cancer.

B cells and antibody-mediated immunity seems to be important in immunity against *Nocardia* spp. In patients with mycetoma, IgG1, 2, 3, 4, and M are higher than those in controls, suggesting an antibody response against *Nocardia* spp. (349). Interestingly, some patients with hematologic malignancies treated whit anti-BTK therapy develop this disease, highlighting the importance of B cells (350). Antibody disorders have also been described in patients with nocardiosis. In 1977, two cases of nocardiosis were described in patients with hypogammaglobulinemia. This disease consists of a reduction in the titers of antibodies and hence impaired immune response (13, 14). Similarly, a nocardiosis patient with common variable immunodeficiency consisting of deficient levels of IgG, IgA, and IgM was reported (18). Finally, one pediatric patient with hyper IgE syndrome, a disease characterized by elevated serum IgE level, chronic dermatitis, intense pruritus, and severe recurrent infection, died due to *Nocardia* infection (351). Despite the mechanisms in which B cells and antibody-mediated responses contribute to immunity to *Nocardia* spp. is not well-understood; the clinical evidence showcases the role of B cell-mediated immunity in fending off *Nocardia* spp.

The observation that patients who have AIDS develop nocardiosis made evident early on that T cells are essential to keep *Nocardia* at bay (22–24). *Nocardia* spp. infection is usually seen in these patients when CD4^+^ counts drop to 35 cells/μl (352). Consistently, two patients with CD4^+^ T cell lymphocytopenia that developed pulmonary and CNS nocardiosis have been reported (353, 354). Similarly, dampened cellular immunity in patients treated with corticosteroids long-term also predisposes to nocardiosis (355). Furthermore, treatments that block cell mediated immunity such as CD3 (OKT3) or CD52 in the treatment of autoimmune and autoinflammatory diseases, have also been shown to predispose to nocardiosis (356–358). Finally, a severe combined immunodeficiency patient lacking B and T cells with a Nocardia infection has been reported (111). These studies confirm the role of T cells in immunity against *Nocardia* and suggest that CD4^+^ T cells play a pivotal role in such immunity.

Specific cytokines have also been implicated in human immunity to *Nocardia* spp. It has been observed that PBMCs from patients with mycetoma caused by *N. brasiliensis* show low IFN-γ production when stimulated with *N. brasiliensis* lysates *ex vivo* but a high concentration of IL-4, IL-10, IL-12, and TNF making the authors suggest at T_H_2 response (359). The widespread use of blocking antibodies against cytokines for therapy of autoimmune and autoinflammatory diseases, confirmed the importance of TNF to fight *Nocardia* spp. Some patients under treatment with anti-TNF antibodies used to treat Crohn's disease (360) or inflammatory bowel disease (361) to develop nocardiosis as a complication. More extensive studies of cohorts of patients with different conditions, all treated with anti-TNF, confirmed that the blockade of TNF causes nocardiosis in a percentage of those patients (362, 363). Anti-IL-12p40 therapy (Blocking both IL-12 and IL-23) also used for inflammatory diseases such as inflammatory bowel disease has been described to cause nocardiosis in a patient from Australia (364). The vital role of these two cytokines, IL-12 and IL-23, in immunity against *Nocardia* spp. was confirmed by studying patients with IL-12Rβ1 and IL-12p40 complete deficiencies. These two deficiencies abolish both IL-12 and IL-23 signaling and are genetic etiologies of MSMD. Patients with MSMD display a selective susceptibility to infection by weakly virulent mycobacteria (26). A group of patients with either IL-12p40 or IL-12Rβ1 complete deficiency has been described to curse with nocardiosis combined with *Mycobacteria, Salmonella*, or *Klebsiella*. This shows the importance of IL-12 and or IL-23 in immunity against *Nocardia* spp. (365–368). Furthermore, in a set of mixed lymphocyte reaction experiments with *N. farcinica*, the authors showed that monocyte-derived DCs co-cultured with T cells are capable of producing high levels of TNF and IL-23 and eliminate engulfed *N. farcinica*. This experiment corroborates the importance of IL-23 and suggests that DCs contribute to fighting *Nocardia* (369). Besides, some nocardiosis patients with, a priori, no additional risk factor, have been found to produce neutralizing anti-GM-CSF auto-antibodies (370). Overall, the combination of clinical, pharmacological, immunological, and genetic evidence indicates that TNF, GM-CSF, IL-12, and or IL-23 are essential players in immunity against *Nocardia* spp.

## Concluding Remarks, Is Nocardiosis a PID?

Although nocardiosis is considered a disease of immunocompromised individuals, the evidence shows that individuals, a priory healthy, can also have this disease. The description of such patients has been growing during recent decades, although it is still considered to be underestimated. In this group of patients, the disease is less severe than in immunocompromised individuals. However, even after appropriate treatment, mortality, and morbidity are high. Sometimes this is due to the late diagnosis and late application of the treatment given that this disease is not considered in the differential diagnosis in patients without risk factors. Besides, TMP-SMX, the most frequently used therapy in this group of patients, has numerous side effects. Therefore, the study of patients with isolated nocardiosis is necessary to better understand the causes and pathophysiology of this disease, which will improve its diagnosis and treatment. During the past two decades, the notion that severe infectious disease in patients otherwise healthy can arise from single-gene inborn errors of immunity has taken steam (27, 28). The genetic dissection of diseases such as MSMD (26), human papillomavirus-associated epidermodysplasia verruciformis (29), invasive pneumococcal disease (371), invasive dermatophytic disease (372), or chronic mucocutaneous candidiasis (32) among others, have shown that mutations in one gene can cause a selective susceptibility to one specific kind of pathogens. That has allowed scientists and physicians to delineate the critical and non-redundant players in immunity against that given pathogen and propose preventive treatment to individuals at risk as well as genetic diagnosis and counseling for patients and families. Nocardiosis shows some similarities with the diseases mentioned above since it is also caused by a weakly virulent pathogen of ubiquitous exposure. Patients with a weakened immune system are more susceptible and given that outbreaks only occur among immunosuppressed individuals shows that exposure to *Nocardia* spp. is necessary, but a host predisposition is also needed. Therefore, isolated nocardiosis may be an undiagnosed PID caused by a single-gene inborn error of immunity that can explain its selective susceptibility to *Nocardia* spp. infection. To test this hypothesis and to try to improve the diagnosis and treatment of this group of patients, we are recruiting patients with isolated nocardiosis to identify the genetic and immunological causes of this disease.

## Author Contributions

RM-B did the literature research, analyzed, and graphed the data and wrote the manuscript.

## Conflict of Interest

The author declares that the research was conducted in the absence of any commercial or financial relationships that could be construed as a potential conflict of interest.
